# *catena*-Poly[copper(II)-bis­(μ-2-formyl-6-meth­oxy­phenolato-κ^4^ *O* ^2^,*O* ^1^:*O* ^1^,*O* ^6^)-[(methanol-κ*O*)sodium]-μ-perchlorato-κ^2^ *O*:*O*′]

**DOI:** 10.1107/S1600536812000876

**Published:** 2012-01-14

**Authors:** Ting Gao, Po Gao, Hong-Feng Li, Ju-Wen Zhang, Li-Li Xu

**Affiliations:** a Key Laboratory of Chemical Engineering Processes & Technology for High-Efficiency Conversion, College of Heilongjiang Province, Heilongjiang University, Harbin 150080, People’s Republic of China

## Abstract

In the title heterodinuclear complex, [CuNa(C_8_H_7_O_3_)_2_(ClO_4_)(CH_3_OH)]_*n*_, the Cu^II^ ion is five-coordinated by four O atoms from two 2-formyl-6-meth­oxy­phenolate anions and one O atom from a perchlorate anion in a distorted square-pyramidal geometry. The Na^+^ ion is six-coordinated by four O atoms from two 2-formyl-6-meth­oxy­phenolate ligands, one O atom of a methanol mol­ecule and one O atom of a perchlorate anion. The perchlorate anions link the Na^+^ and Cu^II^ ions, forming a chain along [010]. O—H⋯O hydrogen bonds connect the chains. π–π inter­actions are present between the benzene rings [centroid–centroid distances = 3.566 (2) and 3.702 (2) Å]. The O atoms of the perchlorate anion are disordered over two sets of sites, with an occupancy ratio of 0.481 (8):0.519 (8).

## Related literature

For related structures, see: Gao *et al.* (2011 ▶); Lin & Zeng (2006 ▶); Yang *et al.* (2012 ▶).
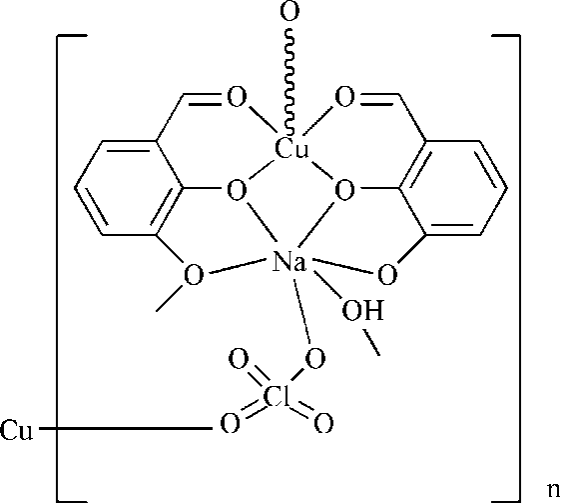


## Experimental

### 

#### Crystal data

[CuNa(C_8_H_7_O_3_)_2_(ClO_4_)(CH_4_O)]
*M* *_r_* = 520.29
Triclinic, 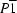

*a* = 7.9552 (16) Å
*b* = 8.9453 (18) Å
*c* = 15.563 (3) Å
α = 81.27 (3)°
β = 84.24 (3)°
γ = 68.25 (3)°
*V* = 1015.6 (4) Å^3^
*Z* = 2
Mo *K*α radiation
μ = 1.29 mm^−1^
*T* = 293 K
0.43 × 0.28 × 0.28 mm

#### Data collection

Rigaku R-AXIS RAPID diffractometer
Absorption correction: multi-scan (*ABSCOR*; Higashi, 1995 ▶) *T*_min_ = 0.610, *T*_max_ = 0.714
9778 measured reflections
4591 independent reflections
3948 reflections with *I* > 2σ(*I*)
*R*_int_ = 0.021

#### Refinement

*R*[*F* ^2^ > 2σ(*F* ^2^)] = 0.034
*wR*(*F* ^2^) = 0.108
*S* = 1.11
4591 reflections
321 parameters
48 restraints
H-atom parameters constrained
Δρ_max_ = 0.58 e Å^−3^
Δρ_min_ = −0.59 e Å^−3^

### 

Data collection: *RAPID-AUTO* (Rigaku, 1998 ▶); cell refinement: *RAPID-AUTO*; data reduction: *CrystalStructure* (Rigaku/MSC, 2002 ▶); program(s) used to solve structure: *SHELXS97* (Sheldrick, 2008 ▶); program(s) used to refine structure: *SHELXL97* (Sheldrick, 2008 ▶); molecular graphics: *DIAMOND* (Brandenburg, 1999 ▶); software used to prepare material for publication: *SHELXTL* (Sheldrick, 2008 ▶).

## Supplementary Material

Crystal structure: contains datablock(s) global, I. DOI: 10.1107/S1600536812000876/hy2501sup1.cif

Structure factors: contains datablock(s) I. DOI: 10.1107/S1600536812000876/hy2501Isup2.hkl

Additional supplementary materials:  crystallographic information; 3D view; checkCIF report

## Figures and Tables

**Table 1:** Hydrogen-bond geometry (Å, °)

*D*—H⋯*A*	*D*—H	H⋯*A*	*D*⋯*A*	*D*—H⋯*A*
O7—H71⋯O10^i^	0.85	2.11	2.874 (7)	149
O7—H71⋯O11′^i^	0.85	2.55	3.334 (13)	154

Symmetry code: (i) 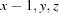.

**Fig. 1.**
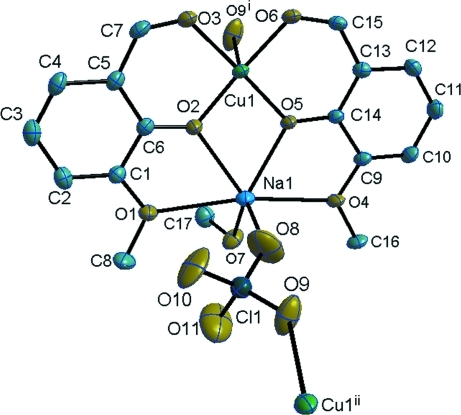
The asymmetric unit of the title compound, showing 30% probability displacement ellipsoids. H atoms and 0.519-occupied O atoms of the perchlorate anion are not shown. [Symmetry codes: (i) x, y-1, z; (ii) x, 1+y, z.]

## supplementary crystallographic information

### Comment

Orthovanillin is a commercial ligand that is able to chelate 3d ions and several structure determinations are known, mainly with copper ions (Lin & Zeng, 2006). Recently, we were interested in the nature of the products obtained by reacting a 3d complex with alkali metal ions (Gao *et al.*, 2011; Yang *et al.*, 2012). In this paper we reacted a Cu complex with sodium perchlorate to yield a heterodinuclear complex. As shown in Fig. 1, the Cu^II^ ion is five-coordinated by two aldehyde O atoms and two phenolate O atoms from two orthovanillin ligands and one O atom from a perchlorate anion in a distorted square-pyramidal geometry. The Cu atom is inserted into the inner cavity surrounded by four O atoms. The Na^+^ ion is ligated by two phenolate O atoms, two methoxyl O atoms, one O atom from a methanol molecule and one O atom from a perchlorate anion. The Cu and Na atoms are bridged by the perchlorate anions, forming a one-dimensional structure along [0 1 0].

### Experimental

To a solution of *o*-vanillin (0.046 g, 0.20 mmol) in dichloromethane (5 ml) was added a solution of copper(II) acetate monohydrate (0.040 g, 0.20 mmol) and sodium perchlorate (0.028 g, 0.20 mmol) in ethanol (5 ml). The mixture was stirred, heated under reflux (30 min) and then allowed to cool to room temperature (yield: 70%). Crystals suitable for X-ray determination were obtained by slow diffusion of diethylether into the solution for one week. Analysis, calculated for C_17_H_18_ClCuNaO_11_: C 39.24, H 3.49%; found: C 39.38, H 3.48%.

### Refinement

H atoms bound to C atoms were placed in calculated positions and treated as riding on their parent atoms, with C—H = 0.93 (aromatic and aldehyde) and 0.96 (methyl) Å and with *U*_iso_(H) = 1.2(1.5 for methyl)*U*_eq_(C). An O-bound H atom was initially located in a differece Fourier map and then treated as a riding atom, with O—H = 0.85 Å and with *U*_iso_(H) = 1.5*U*_eq_(O). The four O atoms of perchlorate anion were disordered over two sets of sites and refined with occupancy factors of 0.481 (8) for O8, O9, O10 and O11 and 0.519 (8) for O8', O9', O10' and O11' atoms. The command 'isor 0.01' was used to restrict the ADP of the above eight O atoms.

### Crystal data

**Table:**

[CuNa(C_8_H_7_O_3_)_2_(ClO_4_)(CH_4_O)]	*Z* = 2
*M**_r_* = 520.29	*F*(000) = 530
Triclinic, *P*1	*D*_x_ = 1.701 Mg m^−^^3^
Hall symbol: -P 1	Mo *K*α radiation, λ = 0.71073 Å
*a* = 7.9552 (16) Å	Cell parameters from 8587 reflections
*b* = 8.9453 (18) Å	θ = 3.0–27.6°
*c* = 15.563 (3) Å	µ = 1.29 mm^−^^1^
α = 81.27 (3)°	*T* = 293 K
β = 84.24 (3)°	Block, green
γ = 68.25 (3)°	0.43 × 0.28 × 0.28 mm
*V* = 1015.6 (4) Å^3^	

### Data collection

**Table:**

Rigaku R-AXIS RAPID diffractometer	4591 independent reflections
Radiation source: rotation anode	3948 reflections with *I* > 2σ(*I*)
graphite	*R*_int_ = 0.021
ω scans	θ_max_ = 27.5°, θ_min_ = 3.0°
Absorption correction: multi-scan (*ABSCOR*; Higashi, 1995)	*h* = −10→10
*T*_min_ = 0.610, *T*_max_ = 0.714	*k* = −11→11
9778 measured reflections	*l* = −20→20

### Refinement

**Table:**

Refinement on *F*^2^	Secondary atom site location: difference Fourier map
Least-squares matrix: full	Hydrogen site location: inferred from neighbouring sites
*R*[*F*^2^ > 2σ(*F*^2^)] = 0.034	H-atom parameters constrained
*wR*(*F*^2^) = 0.108	*w* = 1/[σ^2^(*F*_o_^2^) + (0.0586*P*)^2^ + 0.4273*P*] where *P* = (*F*_o_^2^ + 2*F*_c_^2^)/3
*S* = 1.11	(Δ/σ)_max_ = 0.001
4591 reflections	Δρ_max_ = 0.57 e Å^−^^3^
321 parameters	Δρ_min_ = −0.59 e Å^−^^3^
48 restraints	Extinction correction: *SHELXL97* (Sheldrick, 2008), Fc^*^=kFc[1+0.001xFc^2^λ^3^/sin(2θ)]^-1/4^
Primary atom site location: structure-invariant direct methods	Extinction coefficient: 0.035 (3)

### Special details

**Table:**

Geometry. All e.s.d.'s (except the e.s.d. in the dihedral angle between two l.s. planes) are estimated using the full covariance matrix. The cell e.s.d.'s are taken into account individually in the estimation of e.s.d.'s in distances, angles and torsion angles; correlations between e.s.d.'s in cell parameters are only used when they are defined by crystal symmetry. An approximate (isotropic) treatment of cell e.s.d.'s is used for estimating e.s.d.'s involving l.s. planes.
Refinement. Refinement of *F*^2^ against ALL reflections. The weighted *R*-factor *wR* and goodness of fit *S* are based on *F*^2^, conventional *R*-factors *R* are based on *F*, with *F* set to zero for negative *F*^2^. The threshold expression of *F*^2^ > σ(*F*^2^) is used only for calculating *R*-factors(gt) *etc*. and is not relevant to the choice of reflections for refinement. *R*-factors based on *F*^2^ are statistically about twice as large as those based on *F*, and *R*- factors based on ALL data will be even larger.

### Fractional atomic coordinates and isotropic or equivalent isotropic displacement parameters (Å^2^)

**Table:**

	*x*	*y*	*z*	*U*_iso_*/*U*_eq_	Occ. (<1)
C1	0.4762 (3)	0.6763 (3)	0.08377 (17)	0.0364 (5)	
C2	0.5719 (4)	0.6802 (4)	0.00596 (19)	0.0458 (6)	
H2	0.5263	0.7663	−0.0374	0.055*	
C3	0.7386 (4)	0.5555 (4)	−0.0092 (2)	0.0537 (7)	
H3	0.8026	0.5604	−0.0623	0.064*	
C4	0.8070 (4)	0.4280 (4)	0.0531 (2)	0.0484 (7)	
H4	0.9171	0.3459	0.0424	0.058*	
C5	0.7105 (3)	0.4198 (3)	0.13468 (17)	0.0374 (5)	
C6	0.5417 (3)	0.5456 (3)	0.15163 (16)	0.0341 (5)	
C7	0.7836 (3)	0.2828 (3)	0.1967 (2)	0.0433 (6)	
H7	0.8926	0.2054	0.1797	0.052*	
C8	0.2420 (5)	0.9285 (4)	0.0431 (2)	0.0582 (8)	
H8A	0.3249	0.9853	0.0322	0.087*	
H8B	0.1275	0.9990	0.0651	0.087*	
H8C	0.2256	0.8945	−0.0100	0.087*	
C9	0.0881 (3)	0.6812 (3)	0.48246 (16)	0.0332 (5)	
C10	0.0140 (3)	0.6796 (3)	0.56545 (18)	0.0401 (6)	
H10	−0.0893	0.7658	0.5797	0.048*	
C11	0.0922 (4)	0.5491 (4)	0.62961 (18)	0.0451 (6)	
H11	0.0403	0.5495	0.6859	0.054*	
C12	0.2432 (4)	0.4225 (3)	0.60967 (17)	0.0419 (6)	
H12	0.2955	0.3374	0.6526	0.050*	
C13	0.3218 (3)	0.4197 (3)	0.52328 (17)	0.0344 (5)	
C14	0.2437 (3)	0.5486 (3)	0.45824 (15)	0.0302 (5)	
C15	0.4787 (3)	0.2836 (3)	0.50530 (18)	0.0379 (5)	
H15	0.5227	0.2051	0.5523	0.046*	
C16	−0.1374 (4)	0.9355 (3)	0.4313 (2)	0.0482 (7)	
H16A	−0.2361	0.8969	0.4426	0.072*	
H16B	−0.1606	1.0157	0.3810	0.072*	
H16C	−0.1257	0.9829	0.4807	0.072*	
C17	−0.1080 (5)	0.7533 (5)	0.1549 (3)	0.0673 (9)	
H17A	0.0087	0.6922	0.1305	0.101*	
H17B	−0.1874	0.8128	0.1089	0.101*	
H17C	−0.1579	0.6806	0.1903	0.101*	
Cl1	0.36690 (9)	1.08254 (8)	0.25272 (5)	0.04494 (18)	
Cu1	0.50540 (4)	0.39856 (3)	0.32609 (2)	0.03585 (13)	
Na1	0.19514 (14)	0.77412 (13)	0.26850 (7)	0.0424 (3)	
O1	0.3134 (3)	0.7898 (2)	0.10574 (13)	0.0450 (4)	
O2	0.4454 (2)	0.5493 (2)	0.22418 (12)	0.0426 (4)	
O3	0.7213 (3)	0.2523 (2)	0.27085 (13)	0.0456 (5)	
O4	0.0270 (2)	0.8028 (2)	0.41576 (13)	0.0429 (4)	
O5	0.3060 (2)	0.5559 (2)	0.37745 (11)	0.0372 (4)	
O6	0.5644 (2)	0.2566 (2)	0.43425 (13)	0.0424 (4)	
O7	−0.0898 (3)	0.8613 (3)	0.20606 (16)	0.0603 (6)	
H71	−0.1953	0.9215	0.2223	0.090*	
O8	0.313 (2)	0.9655 (12)	0.2973 (6)	0.134 (4)	0.481 (8)
O9	0.3691 (13)	1.1775 (13)	0.3156 (6)	0.090 (3)	0.481 (8)
O10	0.5238 (9)	1.0233 (14)	0.2027 (7)	0.104 (4)	0.481 (8)
O11	0.2316 (12)	1.1649 (14)	0.1896 (6)	0.134 (4)	0.481 (8)
O8'	0.2175 (7)	1.0358 (9)	0.2565 (7)	0.093 (3)	0.519 (8)
O9'	0.3203 (10)	1.2409 (8)	0.2718 (7)	0.086 (3)	0.519 (8)
O10'	0.4646 (19)	1.0763 (15)	0.1746 (6)	0.140 (4)	0.519 (8)
O11'	0.5004 (15)	0.9750 (12)	0.3065 (8)	0.164 (5)	0.519 (8)

### Atomic displacement parameters (Å^2^)

**Table:**

	*U*^11^	*U*^22^	*U*^33^	*U*^12^	*U*^13^	*U*^23^
C1	0.0334 (11)	0.0377 (12)	0.0389 (13)	−0.0130 (10)	−0.0007 (10)	−0.0073 (10)
C2	0.0491 (15)	0.0542 (16)	0.0385 (14)	−0.0258 (13)	0.0003 (12)	−0.0023 (12)
C3	0.0503 (16)	0.070 (2)	0.0445 (15)	−0.0287 (15)	0.0133 (13)	−0.0103 (14)
C4	0.0365 (13)	0.0568 (17)	0.0508 (16)	−0.0156 (12)	0.0138 (12)	−0.0175 (13)
C5	0.0322 (11)	0.0395 (13)	0.0410 (14)	−0.0121 (10)	0.0045 (10)	−0.0126 (10)
C6	0.0318 (11)	0.0336 (12)	0.0370 (12)	−0.0113 (10)	0.0014 (10)	−0.0087 (10)
C7	0.0299 (11)	0.0374 (13)	0.0552 (16)	−0.0022 (10)	0.0055 (11)	−0.0140 (12)
C8	0.0620 (19)	0.0440 (16)	0.0535 (18)	−0.0038 (14)	−0.0136 (15)	0.0062 (13)
C9	0.0264 (10)	0.0290 (11)	0.0407 (13)	−0.0062 (9)	0.0004 (9)	−0.0047 (9)
C10	0.0316 (11)	0.0404 (13)	0.0439 (14)	−0.0077 (10)	0.0057 (10)	−0.0111 (11)
C11	0.0447 (14)	0.0531 (16)	0.0359 (13)	−0.0170 (12)	0.0050 (11)	−0.0076 (11)
C12	0.0482 (14)	0.0424 (14)	0.0327 (12)	−0.0156 (12)	−0.0048 (11)	0.0017 (10)
C13	0.0321 (11)	0.0312 (11)	0.0384 (13)	−0.0103 (9)	−0.0040 (10)	−0.0015 (9)
C14	0.0259 (10)	0.0265 (10)	0.0351 (12)	−0.0063 (9)	−0.0003 (9)	−0.0037 (9)
C15	0.0342 (12)	0.0293 (11)	0.0428 (13)	−0.0052 (10)	−0.0073 (10)	0.0053 (10)
C16	0.0365 (13)	0.0316 (13)	0.0631 (18)	0.0043 (11)	−0.0030 (12)	−0.0086 (12)
C17	0.074 (2)	0.075 (2)	0.060 (2)	−0.033 (2)	−0.0058 (18)	−0.0119 (18)
Cl1	0.0447 (3)	0.0444 (4)	0.0470 (4)	−0.0179 (3)	0.0014 (3)	−0.0071 (3)
Cu1	0.03095 (18)	0.02748 (18)	0.0382 (2)	0.00017 (12)	0.00257 (12)	−0.00201 (12)
Na1	0.0378 (5)	0.0354 (5)	0.0444 (6)	−0.0028 (4)	−0.0055 (4)	−0.0013 (4)
O1	0.0428 (10)	0.0383 (10)	0.0424 (10)	−0.0039 (8)	−0.0028 (8)	0.0017 (8)
O2	0.0382 (9)	0.0373 (9)	0.0375 (9)	0.0003 (7)	0.0040 (8)	−0.0005 (7)
O3	0.0391 (9)	0.0340 (9)	0.0501 (11)	0.0003 (8)	0.0036 (8)	−0.0033 (8)
O4	0.0375 (9)	0.0287 (8)	0.0461 (10)	0.0041 (7)	0.0032 (8)	−0.0005 (7)
O5	0.0335 (8)	0.0295 (8)	0.0352 (9)	0.0016 (7)	0.0038 (7)	−0.0006 (7)
O6	0.0392 (9)	0.0285 (9)	0.0461 (10)	0.0017 (7)	−0.0020 (8)	−0.0006 (7)
O7	0.0401 (10)	0.0648 (14)	0.0727 (15)	−0.0083 (10)	−0.0068 (10)	−0.0239 (12)
O8	0.216 (9)	0.107 (6)	0.119 (6)	−0.117 (6)	−0.013 (6)	0.018 (5)
O9	0.090 (5)	0.121 (7)	0.091 (5)	−0.064 (5)	0.026 (4)	−0.064 (5)
O10	0.035 (3)	0.137 (7)	0.124 (7)	0.007 (3)	0.012 (3)	−0.073 (6)
O11	0.100 (5)	0.169 (8)	0.097 (5)	−0.007 (5)	−0.034 (4)	−0.001 (5)
O8'	0.046 (3)	0.078 (4)	0.174 (7)	−0.031 (3)	0.009 (3)	−0.059 (5)
O9'	0.059 (3)	0.049 (3)	0.157 (7)	−0.021 (3)	0.013 (4)	−0.038 (4)
O10'	0.193 (10)	0.154 (8)	0.071 (5)	−0.077 (7)	0.044 (6)	−0.005 (5)
O11'	0.165 (7)	0.126 (6)	0.171 (7)	−0.033 (5)	−0.086 (6)	0.073 (6)

### Geometric parameters (Å, °)

**Table:**

C1—O1	1.367 (3)	C16—O4	1.432 (3)
C1—C2	1.367 (4)	C16—H16A	0.9600
C1—C6	1.427 (4)	C16—H16B	0.9600
C2—C3	1.407 (4)	C16—H16C	0.9600
C2—H2	0.9300	C17—O7	1.396 (4)
C3—C4	1.360 (5)	C17—H17A	0.9600
C3—H3	0.9300	C17—H17B	0.9600
C4—C5	1.422 (4)	C17—H17C	0.9600
C4—H4	0.9300	Cl1—O8	1.347 (7)
C5—C7	1.413 (4)	Cl1—O10	1.373 (7)
C5—C6	1.428 (3)	Cl1—O10'	1.373 (9)
C6—O2	1.297 (3)	Cl1—O11'	1.390 (8)
C7—O3	1.242 (3)	Cl1—O8'	1.392 (5)
C7—H7	0.9300	Cl1—O9	1.395 (8)
C8—O1	1.426 (3)	Cl1—O9'	1.396 (7)
C8—H8A	0.9600	Cl1—O11	1.439 (8)
C8—H8B	0.9600	Cu1—O5	1.8890 (18)
C8—H8C	0.9600	Cu1—O2	1.8941 (19)
C9—C10	1.365 (4)	Cu1—O6	1.932 (2)
C9—O4	1.365 (3)	Cu1—O3	1.944 (2)
C9—C14	1.426 (3)	Cu1—O9^i^	2.614 (11)
C10—C11	1.407 (4)	Cu1—O9'^i^	2.650 (8)
C10—H10	0.9300	Cu1—Na1	3.4010 (17)
C11—C12	1.358 (4)	Na1—O5	2.342 (2)
C11—H11	0.9300	Na1—O8	2.349 (9)
C12—C13	1.424 (4)	Na1—O7	2.365 (2)
C12—H12	0.9300	Na1—O2	2.379 (2)
C13—C14	1.408 (3)	Na1—O8'	2.390 (7)
C13—C15	1.423 (3)	Na1—O4	2.533 (2)
C14—O5	1.306 (3)	Na1—O1	2.614 (2)
C15—O6	1.246 (3)	O7—H71	0.8500
C15—H15	0.9300		
			
O1—C1—C2	125.9 (3)	O11'—Cl1—O8'	111.4 (6)
O1—C1—C6	113.0 (2)	O8—Cl1—O9	104.4 (6)
C2—C1—C6	121.1 (2)	O10—Cl1—O9	116.6 (6)
C1—C2—C3	120.7 (3)	O10'—Cl1—O9'	105.4 (7)
C1—C2—H2	119.6	O11'—Cl1—O9'	110.6 (6)
C3—C2—H2	119.6	O8'—Cl1—O9'	112.5 (4)
C4—C3—C2	120.5 (3)	O8—Cl1—O11	104.6 (7)
C4—C3—H3	119.7	O10—Cl1—O11	103.6 (6)
C2—C3—H3	119.7	O9—Cl1—O11	113.9 (6)
C3—C4—C5	120.1 (3)	O5—Cu1—O2	83.82 (8)
C3—C4—H4	119.9	O5—Cu1—O6	93.45 (8)
C5—C4—H4	119.9	O2—Cu1—O6	176.21 (8)
C7—C5—C4	118.3 (2)	O5—Cu1—O3	174.49 (8)
C7—C5—C6	121.4 (2)	O2—Cu1—O3	93.50 (9)
C4—C5—C6	120.2 (3)	O6—Cu1—O3	89.00 (8)
O2—C6—C1	118.2 (2)	O2—Cu1—O9^i^	107.1 (2)
O2—C6—C5	124.5 (2)	O3—Cu1—O9^i^	84.5 (2)
C1—C6—C5	117.2 (2)	O5—Cu1—O9^i^	100.9 (2)
O3—C7—C5	128.4 (2)	O6—Cu1—O9^i^	75.9 (2)
O3—C7—H7	115.8	O2—Cu1—O9'^i^	88.9 (2)
C5—C7—H7	115.8	O3—Cu1—O9'^i^	87.86 (19)
O1—C8—H8A	109.5	O5—Cu1—O9'^i^	96.87 (19)
O1—C8—H8B	109.5	O6—Cu1—O9'^i^	94.0 (2)
H8A—C8—H8B	109.5	O5—Na1—O8	104.4 (3)
O1—C8—H8C	109.5	O5—Na1—O7	126.05 (9)
H8A—C8—H8C	109.5	O8—Na1—O7	120.1 (3)
H8B—C8—H8C	109.5	O5—Na1—O2	64.72 (7)
C10—C9—O4	125.8 (2)	O8—Na1—O2	106.8 (4)
C10—C9—C14	120.8 (2)	O7—Na1—O2	121.84 (9)
O4—C9—C14	113.4 (2)	O5—Na1—O8'	127.7 (2)
C9—C10—C11	120.7 (2)	O8—Na1—O8'	24.3 (3)
C9—C10—H10	119.6	O7—Na1—O8'	96.63 (19)
C11—C10—H10	119.6	O2—Na1—O8'	120.27 (15)
C12—C11—C10	120.3 (2)	O5—Na1—O4	63.79 (7)
C12—C11—H11	119.9	O8—Na1—O4	87.9 (3)
C10—C11—H11	119.9	O7—Na1—O4	87.65 (8)
C11—C12—C13	120.2 (2)	O2—Na1—O4	128.46 (8)
C11—C12—H12	119.9	O8'—Na1—O4	93.2 (2)
C13—C12—H12	119.9	O5—Na1—O1	126.62 (8)
C14—C13—C15	121.7 (2)	O8—Na1—O1	92.0 (3)
C14—C13—C12	120.1 (2)	O7—Na1—O1	82.97 (9)
C15—C13—C12	118.2 (2)	O2—Na1—O1	61.92 (7)
O5—C14—C13	124.3 (2)	O8'—Na1—O1	82.4 (2)
O5—C14—C9	117.9 (2)	O4—Na1—O1	169.07 (7)
C13—C14—C9	117.9 (2)	C1—O1—C8	116.7 (2)
O6—C15—C13	128.0 (2)	C1—O1—Na1	118.00 (15)
O6—C15—H15	116.0	C8—O1—Na1	123.66 (18)
C13—C15—H15	116.0	C6—O2—Cu1	127.00 (16)
O4—C16—H16A	109.5	C6—O2—Na1	126.71 (16)
O4—C16—H16B	109.5	Cu1—O2—Na1	104.90 (9)
H16A—C16—H16B	109.5	C7—O3—Cu1	124.61 (17)
O4—C16—H16C	109.5	C9—O4—C16	117.8 (2)
H16A—C16—H16C	109.5	C9—O4—Na1	118.86 (14)
H16B—C16—H16C	109.5	C16—O4—Na1	123.15 (17)
O7—C17—H17A	109.5	C14—O5—Cu1	127.46 (15)
O7—C17—H17B	109.5	C14—O5—Na1	125.99 (14)
H17A—C17—H17B	109.5	Cu1—O5—Na1	106.52 (8)
O7—C17—H17C	109.5	C15—O6—Cu1	125.08 (16)
H17A—C17—H17C	109.5	C17—O7—Na1	114.4 (2)
H17B—C17—H17C	109.5	C17—O7—H71	108.0
O8—Cl1—O10	113.3 (8)	Na1—O7—H71	132.5
O10'—Cl1—O11'	100.0 (8)	Cl1—O8—Na1	138.4 (7)
O10'—Cl1—O8'	116.1 (7)	Cl1—O8'—Na1	131.5 (4)

Symmetry codes: (i) *x*, *y*−1, *z*.

### Hydrogen-bond geometry (Å, °)

**Table:**

*D*—H···*A*	*D*—H	H···*A*	*D*···*A*	*D*—H···*A*
O7—H71···O10^ii^	0.85	2.11	2.874 (7)	149
O7—H71···O11'^ii^	0.85	2.55	3.334 (13)	154

Symmetry codes: (ii) *x*−1, *y*, *z*.
